# Application of metabolomics to drug discovery and understanding the mechanisms of action of medicinal plants with anti-tuberculosis activity

**DOI:** 10.1186/s40169-018-0208-3

**Published:** 2018-10-01

**Authors:** Naasson Tuyiringire, Deusdedit Tusubira, Jean-Pierre Munyampundu, Casim Umba Tolo, Claude M. Muvunyi, Patrick Engeu Ogwang

**Affiliations:** 1 Pharm-BioTechnology and Traditional Medicine Centre (PHARMBIOTRAC), Mbarara University of Science & Technology, P.O. Box, 1410 Mbarara, Uganda; 2 School of Science, College of Science and Technology, University of Rwanda, Avenue de l’Armée, P.O. Box 3900, Kigali, Rwanda; 3 College of Medicine and Health Sciences, University of Rwanda, University Avenue, P.O. Box 56, Butare, Rwanda; 4 Department of Biomedicine, University of Bergen, Jonas Lies Vei 91, 5020 Bergen, Norway

**Keywords:** *Mycobacterium tuberculosis* (*M.tb*), Traditional medicinal plants, Metabolomics, Antimycobacterial, Multidrug resistance

## Abstract

Human tuberculosis (TB) is amongst the oldest and deadliest human bacterial diseases that pose major health, social and economic burden at a global level. Current regimens for TB treatment are lengthy, expensive and ineffective to emerging drug resistant strains. Thus, there is an urgent need for identification and development of novel TB drugs and drug regimens with comprehensive and specific mechanisms of action. Many medicinal plants are traditionally used for TB treatment. While some of their phytochemical composition has been elucidated, their mechanisms of action are not well understood. Insufficient knowledge on *Mycobacterium tuberculosis* (*M.tb*) biology and the complex nature of its infection limit the effectiveness of current screening-based methods used for TB drug discovery. Nonetheless, application of metabolomics tools within the ‘omics’ approaches, could provide an alternative method of elucidating the mechanism of action of medicinal plants. Metabolomics aims at high throughput detection, quantification and identification of metabolites in biological samples. Changes in the concentration of specific metabolites in a biological sample indicate changes in the metabolic pathways. In this paper review and discuss novel methods that involve application of metabolomics to drug discovery and the understanding of mechanisms of action of medicinal plants with anti-TB activity. Current knowledge on TB infection, anti-TB drugs and mechanisms of action are also included. We further highlight metabolism of *M. tuberculosis* and the potential drug targets, as well as current approaches in the development of anti-TB drugs.

## Background

Infection with *Mycobacterium tuberculosis* (*M.tb*) results in tuberculosis (TB) [1], a pandemic disease [2] that mostly affects developing countries [3]. According to World Health Organization (WHO) 9.6 million new cases of the disease were reported in 2015 alone, with over 2 million deaths in the same year [4]. Tuberculosis either kills the infected individual or renders him/her incapable of assuming normal functions. Upon gaining entry into a new host, *M.tb* may result into an active infection or remain latent [5]. In the WHO global Tuberculosis report of 2014, it was estimated that one-third of world population is latently infected with TB [1, 3]. Reactivation of latent TB in immune deficit people such as human immunodeficiency virus (HIV)-positive individuals [6] results into active TB [1, 5, 6]. Thus, TB is a leading killer in HIV-infected individuals [7, 8]. A third of HIV death is caused by TB [9].

**Fig. 1:**
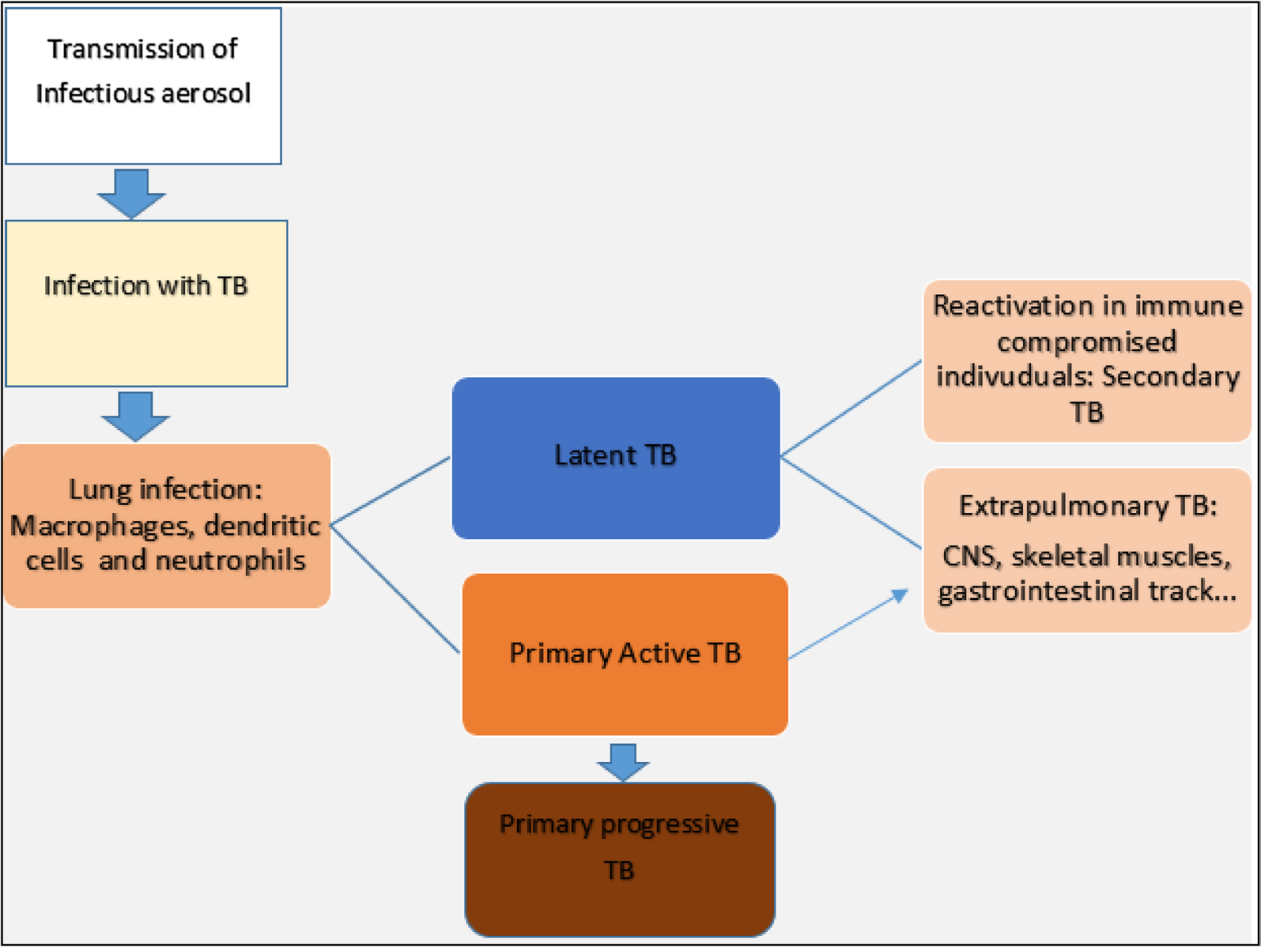
Tuberculosis infection and development: after the transmission with infectious aerosol, the *M.tb* bacilli infect the lung macrophages, dendritic cells and neutrophils. The infection may result in active or latent. The latent and active infection can be spread to extrapulmonary organs. The reactivation of latent infection results in secondary TB

*Mycobacterium tuberculosis* is spread via infectious aerosols from infected person (Fig. 1).

Bacilli in aerosols inhaled deep into lung alveoli lodge in terminal air spaces [1, 6]. Infection starts when *M.tb* interacts with host target cells [10]. Target cells are mainly macrophages, dendritic cells and recruited neutrophils [5]. The bacteria are engulfed by phagocytosis, in attempt to destroy invading pathogens by the host cells. As an intracellular pathogen, living in a hostile environment of immune cells, *M.tb* evolved means to survive. Mycobacteria are able to block normal acidification of phagosomes by preventing phagolysosomal fusion [1, 5, 11].

This enables the *M.tb* bacilli to establish a niche where they replicate within inactivated macrophages. Such macrophages offer limited defense to the host [6].

Fortunately bacterial replication is slowed following recruitment of innate components when host effectors like reactive oxygen species (ROS) and nitrogen intermediates are released. Innate immunity is strengthened by cellular immunity activated through release of chemokines such as interferon-gamma, tumor necrosis factor, interleukins and tumor growth factors. Immune activation results in recruitment of lymphocytes and additional macrophages that assemble into a granuloma [1, 2, 7]. A granuloma is described as a multicellular structure that sequesters the infecting bacteria from the surrounding tissue, and further inhibits the rate of bacilli replication [1, 12]. The interior of the granuloma has been described as necrotic (or “caseous”) and hypoxic [2]. At this stage bacilli are thought to enter a quiescent (latent) state characterized by limited or no replication and phenotypic resistance to drugs [13]. In the granuloma, although actively replicating bacilli are found, non-replicating persistent (NRP) (dormant) forms of *M.tb* can also be found. The latter are induced by the environmental conditions that are found in specific granuloma types, particularly those associated with hypoxia/anoxia, nutrient deprivation and nitric oxide production [14].

The NRP state of *M.tb* is characterized by the presence of non-dividing bacilli with low-metabolic state, a phenotype associated with resistance/tolerance to standard anti-TB agents [1, 6, 15]. Thus novel anti-TB drugs that can target and kill the bacilli inside the granuloma are likely to offer the best opportunity to reduce treatment length and eliminate relapse [1, 16, 17]. Resistance is also attributable to *M.tb*’s unique obligate intracellular parasitic biology [1, 6, 18, 19]. To cope with this hostile environment, *M.tb* has evolved specific metabolic profiles not present in host cells [20]. Fortunately, metabolic reprogramming could be an opportunity for drug targeting as will be explained later.

Infection with TB is not limited to lungs only. Bacilli may be localized to different compartments/tissues of the host on spread through blood and lymphatics [5, 11]. The outcome may either be active or latent infections with TB. Activation of cellular immunity may result into containment of over 90–95% of bacterial replication [1, 5]. Therefore, the state of disease (active infection) and the latency are defined by the state of host immunity. In fact, during latent infection, a dynamic equilibrium between the bacilli and host immune responses is established. Any event that weakens cell mediated immunity may lead to; active bacterial replication, tissue damage and disease discussed in background [1, 5]. TB pathogenesis has been extensively reviewed by Salvatore and Zhang [6]. In short, it includes: Primary tuberculosis, primary progressive TB, Secondary TB and extra pulmonary TB [5]. Most common sites of extra pulmonary disease are the pleura, secondary lymphatic tissues, genitourinary tract, skeletal system, gastrointestinal tract, meninges and central nervous system (CNS). The most important extra pulmonary TB include; central nervous system and miliary TB. Pathological characteristics of TB disease are not mediated by bacterial toxins. Instead they reflect host immune response to mycobacterial antigens [5, 6].

*Mycobacterium tuberculosis* virulence factors that trigger immune responses are related to the structure and metabolism of the bacterium. These include: mycobacterial cell surfaces that mediate the interactions with the host receptors, inhibition of phagosomal maturation and phagosome–lysosome fusion, mitigation of oxidative stress, mycobacterial secretion systems, and bacterial proteasome [2, 3, 5, 6]. Those virulence factors along with the others that are discussed later are regarded as potential anti-TB drug targets.

Since its discovery in 1882 [1, 8, 9], efforts have been made to treat or prevent *M.tb* infection, but there are still gaps to be bridged for its effective treatment. The association of high TB burden with HIV and the increasing emergence of multidrug resistant (MDR), extended drug resistant (XDR), and total drug resistant (TDR)-TB have worsened the situation [11]. For instance, resistance to rifampin, a TB drug was reported to be associated with the reduction of the efficacy of some antiretroviral drugs [10]. McIlleron et al. [10] demonstrated cross-resistance between rifampin and most commonly used antiretroviral agents through induction of cytochrome-P450 enzymes in patients with HIV-TB co-infection. Rifampin is a promiscuous inducer of expression of a broad array of enzymes and drug-transporting molecules through its activation of a master transcriptional regulator, pregnane X receptor. Induction of cytochrome P-450 enzymes and *P*-glycoprotein by rifampin results in reduced concentrations of non-nucleoside reverse transcriptase inhibitors, particularly protease inhibitors. This potentially results in the loss of antiviral efficacy and development of viral resistance [7, 10].

Tuberculosis was first treated with streptomycin over 73 years ago [12, 13]. At present, so many new drugs have been introduced [21]. However, *M.tb* remains as one of the leading infectious diseases worldwide [9] mostly due to lack of effective anti-TB drugs. Most antimycobacterials are not very effective due to highly adaptable intracellular parasitic biology of *M.tb* as highlighted before.

Almost 80% of the population in developing countries rely on traditional medicine (TM) for their primary healthcare [22]. The reasons for the use of TM include its affordability and accessibility [22, 23]. Unfortunately, the mechanism of action of most medicinal plants traditionally used for TB treatment is not well understood.

Most of the current anti TB drug targets and mechanism of action were identified through target structure-based radiolabeling and screening [18]. Target macromolecules and their role in *M.tb* metabolism can be identified through directed mutagenesis methods [18, 24]. Macromolecule structures of targets can be elucidated by X-ray crystallography or NMR spectroscopy [6, 18]. These methods however are expensive and time consuming usually requiring first knowing the active compound and then radiolabeling it. For medicinal plants extracts usually the active compound may not be known or even where they are know they may work in synergy to kill *M.Tb*. In such a case radiolabeling can difficult at the extract screening phase.

Metabolomics is a recent and emerging field within the ‘omics’ [25–27] science. It is defined as the study of the complete set of metabolites inside cells, tissues, organs and biological fluids. It represents a major and rapidly evolving component of systems biology; a new integrative approach to deciphering the complexity of biological systems [26, 28–30]. It uses advanced analytical techniques such as nuclear magnetic resonance (NMR), liquid chromatography mass spectrometry (LC–MS), gas chromatography–mass spectrometry (GC–MS) or Capillary electrophoresis–mass spectrometry (CE–MS) to comprehensively identify and quantify a large number of metabolites [25, 31]. These techniques have shown great impact on classifying phenotypes, investigation of physiological status, diagnosing disease, measuring the response to treatment, and discovery of biomarkers [32]. The physiological effects of small molecules make the field of metabolomics critical to the aims and interests of many researchers in the pharmaceutical industry [24, 33–36]. Thus, metabolomics is increasingly finding applications that cover the full length of drug discovery and development pipeline i.e. from lead compound discovery to post-approval drug surveillance [36].

Though metabolomics has been used in studying *M.tb* metabolism in general [20], available literature does not indicate its application to understanding anti-TB drug mechanism of traditional medicinal plants. This review aims at exploring new available methodologies for understanding the mechanism of action of anti-TB traditional medicinal plants.

## Current anti-tuberculosis drugs and their mechanism of action

Presently, major antitubercular drugs include, Isoniazid, rifampin, pyrazinamide, ethambutol etc. [7]. Isoniazid was discovered in 1952 [37]. This drug followed streptomycin and paraaminosalicylic acid (PAS) which are usually referred to as the first clinical antibiotics. Isoniazid (INH; isonicotinic acid hydrazide), structurally known as pyridine-4-carboxy hydrazide has been the most commonly used anti-TB drug since its discovery in 1952 [13]. This drug was discovered by three different pharmaceutical companies, BAYER (Leverkusen, Germany); Hoffmann LaRoche (Nutley, NJ, USA) and ER Squibb & Sons (Princeton, NJ, USA) [37], and was shown to inhibit the synthesis of cell envelope of *M.tb* [38]. Considering the importance of the cell membrane in pathogenesis of *M.tb*, INH cured many patients and was dubbed “magic drug” [37]. Since 1952, INH has remained an essential drug in the fight against TB. Isoniazid is a prodrug that requires activation by *M.tb* catalase-peroxidase katG enzyme to form an INH-NAD complex. It inhibits nicotinamide adenine dinucleotide (NADH)-dependent enoyl-ACP reductase (encoded by inhA gene) of the fatty acid synthase type II system, a key player in the mycolic acid biosynthetic pathway of *M.tb* [39]. The inhibition of enoyl-ACP reductase (encoded by inhA gene) causes accumulation of long-chain fatty acids and cell death [38]. In clinical isolates, mutations in the katG and inhA genes have been shown to contribute approximately 70% and 80%, respectively, to isoniazid resistance in *M.tb* [38, 40]. As a prodrug, its activity is importantly influenced by mutations in the katG enzyme. Thus, designing drugs that do not require the katG enzyme activation, but targeting inhA enzyme would be a reasonable alternative to bypass this Isoniazid-associated resistance mechanism. For instance, triclosan inhibits inhA enzyme [41], but it has not been successfully used as anti-TB drug because of its sub-optimal bioavailability [42]. Ethionamide (ETH, 2-ethylisonicotinamide), a derivative of isonicotinic acid, has been used as an anti-TB agent since 1956. The drug has the same mechanism of action as INH. As stated above, *p*-amino salicylic acid (PAS) was one of the first antibiotics to show anti-TB activity. However, its mechanism of action was never elucidated, though it is thought to act through inhibition of thymine synthesis [21].

*Pyrazinamide* (*PZA*) is an analog/derivative of nicotinamide [43]. This is also a prodrug that requires conversion to pyrazinoic acid by *M.tb* pyrazinamidase [encoded by the pyrazinamidase/nicotinamidase (pncA) gene] [37, 44]. The cellular target of PZA was identified as ribosomal protein S1 (RpsA) [45, 46]. As such, it was reported that over-expression of RpsA (wild-type RpsA) is implicated in PZA resistance in *M.tb*. Though the mechanism of action of PZA is not well understood, it is known that binding of activated pyrazinoic acid to RpsA interferes with its binding to the messenger RNA, thus compromising accurate protein synthesis. Some PZA-resistant *M.tb* strains without mutations in pncA have mutations in RpsA [47]. It was found that the RpsA enzyme is essential for translation [45], with its C terminus being involved in trans–translation [37, 48] in *M.tb*. The authors reported that trans–translation in *M.tb* is dispensable during active growth conditions, and also required by some bacterial strains for survival under stress conditions and in disease progression. Thus, the inhibition of particular essential the trans–translation steps in *M.tb* may interfere with its survival under the dormancy state. Moreover, this could possibly explain the ability of diverse stress conditions such as starvation, acidic pH and hypoxia to increase PZA activity against *M.tb* [41, 47, 49, 50].

*Ethambutol* (*EMB*), interferes with mycobacterial cell wall synthesis by inhibiting polymerization of arabinogalactan, an important cell wall component in *M.tb* [21, 51, 52]. It also interrupts utilization of arabinose donor by inhibiting either arabinosyltransferase enzymatic activity or formation of an arabinose acceptor in mycobacteria [53]. The embCAB operon is responsible for ethambutol resistance in *M.tb* [37, 51, 52]. It is worth noting that ethambutol and benzothiazinones block/inhibit the same pathway, although the steps are different.

*Rifampicins* (*RIF*) comprise a group of antibacterial drugs that include the derivatives: rifampicin, rifapentine, rifabutin and rifalazil [41]. These bind bacterial beta RNA polymerase (rpoB) subunit thus interfering with transcription [41]. Resistance to rifampicins in *M.tb* is conferred by mutations in the 81-bp region of the rpoB gene (encoding beta Ribonucleic Acid, RNA polymerase) [54, 55]. Both rifampicin and isoniazid are essential and commonly used as first-line drugs for TB therapy [37]. Following introduction of rifampicin into clinical use, treatment of active TB was reduced from 9–12 to 6 months, while the duration for treatment of latent TB was reduced from 9 to 3 months [40]. It is important to note that rifampicins are among the few drugs that can kill the dormant (non-replicating) strains of *M.tb*. Rifampicin was developed by blind whole-cell screening in an extensive program of chemical modification of the rifamycins, the natural metabolites of *Amycolatopsis mediterranei* [56, 57]. Since rpoB is an essential gene in *M.tb* and RNA polymerase is a proven target for antibacterial and anti-TB therapy, it would be interesting to search for new RNA polymerase inhibitors binding at sites different from that utilized by rifampicin [58].

*Streptomycin* (*SM*) SM is an aminocyclitol glycoside antibiotic. As mentioned in the background, it was the first drug to be used in treatment of TB in 1948. It binds to small 16S rRNA of the 30S subunit of the bacterial ribosome, interfering with the binding of formyl-methionyl-tRNA to the 30S subunit. This leads to the interruption of the initiation of protein synthesis. Other aminoglycosides (kanamycin, amikacin and capreomycin) also inhibit protein synthesis of *M.tb* [21, 59]. As for rifampicins, gaps exist in detecting the metabolites affected my streptomycin.

*Fluoroquinolones* (*FQs*) These include ciprofloxacin, ofloxacin, levofloxacin, and moxifloxacin. They have broad-spectrum anti-bacterial activity. Fluoroquinolones have excellent in vitro and in vivo activity against *M.tb*. They target *M.tb* DNA gyrase, a type II topoisomerase consisting of A and B subunits encoded by *gyrA* and *gyrB* genes, respectively. The DNA gyrase (gyrase) enzyme catalyzes formation of negative supercoils that aid DNA unwinding. Thus, FQs compromise bacterial DNA replication [21, 60]. This results in abnormally localized, condensed chromosomes that blocks DNA replication and interrupts chromosomes segregation [61].

*Cycloserine* (*CS*) CS prevents tuberculosis bacteria from making peptidoglycans needed in the formation of bacterial cell wall. Cycloserine can inhibit the d-alanine racemase enzyme [62]. d-alanine racemase is an isomerase enzyme that catalyzes the conversion of l-alanine to d-alanine. The latter takes part in synthesis of peptidoglycan. Therefore, CS can indirectly inhibit the synthesis of peptidoglycan [43, 63]. Recently, two anti TB drugs (Bedaquiline and Delamanid) were reported [18].

*Bedaquiline* (*TMC-207*) In December 2012, diarylquinoline was approved by FDA as part of combination therapy for treatment of adult patients affected by MDR-TB. It was in phase III of clinical development in 2015 [37] and in confirmatory phase III trials this year [64]. Bedaquiline is considered to be the first major drug approved by FDA for TB therapy in the last four decades. It came out of phenotypic screening of compounds against *M.tb*. The target of bedaquiline was identified through the whole-genome sequencing of *M.tb* and *M. smegmatis* spontaneous mutants that were resistant to diarylquinolines. The resistant mutants showed missense mutations in the atpE gene (encoding the c subunit of ATP synthase) [65]. It was later shown that Bedaquiline acts by inhibiting adenosine triphosphate (ATP) synthase and has activity against active and dormant *M.tb* strains [66]. Human mitochondrial ATP synthase is 20,000-fold less sensitive to diarylquinoline compared to that of the mycobacteria. For this reason ATPase has a high potential as a drug target against *M.tb* [65].

*Delamanid* was reported as a newer mycobacterial cell wall synthesis inhibitor [67]. It inhibits the synthesis of mycobacterial cell wall components, methoxymycolic acid and ketomycolic acid [67]. Delamanid is a pro-drug which is activated by the enzyme deazaflavin dependent nitroreductase (Rv3547) [67]. A reactive intermediate metabolite formed between delamanid and desnitro-imidazooxazole derivative, plays a vital role in the inhibition of mycolic acid production and this explains its mode of action. This drug has received conditional approval from European Medicines Agency (EMA) for treatment of MDR-TB [67]. Preclinical and clinical studies have shown that delamanid has high potency, very low risk for drug–drug interactions and better tolerability [67]. Eight drugs in phase 2–3 trials were reported as current drug pipeline for treatment of TB. They include Bedaquiline and delamanid for confirmatory trials. These two were approved as stated above. The remaining six are in trials. Two of the six (sutezolid and pretomanid) are new compounds. Others are members of rifampicins (rifapentine) and fluoroquinolones (levofloxacin and moxifloxacin) [64]. Table 1 illustrates some of the new drugs and their mechanism of action.

**Table 1:** Summary of representing some of the new drugs and their functions

Anti-TB drugs	Suggested/confirmed function/mechanism of action	References
Pretomanid (PA-824)	1. Potentially act on mycolic acid biosynthetic pathway through depletion of ketoymycolates and accumulation of hydroxymycolates on replicating bacteria	[94]
	2. Des-nitroimidazole derivative from pretomanid metabolism was responsible for generation of reactive nitrogen species and ATP depletion, which would explain its activity under anaerobic conditions	[95]
Delamanid (OPC-67683)	Inhibits synthesis of mycobacterial cell wall components, methoxymycolic acid and ketomycolic acid	[67]
Bedaquiline	Inhibits ATP synthase	[96]
Rifapentine	Inhibits transcription by interacting with mycobacterial RNA polymerase. It does not inhibit the mammalian enzyme	[97]
Linezolid	It is an oxazolidinone. It is a candidate for MDR TB. It inhibits the initiation of the protein synthesis	[98]
Sutezolid	It is an oxazolidinone, an analog of linezolid. It inhibits the protein synthesis	[99]
Levofloxacin and Moxifloxacin	These are members of fluoroquinolones. They inhibit DNA replication by binding bacterial gyrase	[60, 100]

## *Mycobacterium tuberculosis* metabolism and metabolic related potential drug targets

In bacteria, metabolism is commonly used to describe the full set of complex and interconnected chemical transformations that enable individual cells to survive and replicate [68]. It includes pathways that enable the bacteria to respond to the dynamic environments and maintain structural integrity in the face of fluctuating nutrient availability. As such, in an obligate intracellular pathogen such as *M.tb* whose entire life cycle is driven in the context of human infection, metabolism underpins both physiology and pathogenesis [6, 68]. Research on *M.tb* metabolism was mostly done through experimental mycobacteriology. It involved use of genetic tools to disrupt enzymatic and regulatory functions. This has provided critical insights into the metabolic pathways that are essential for mycobacterial survival and pathogenesis [6, 20, 69]. Despite the efforts to understand TB metabolism and its use to identify potential drugs targets, it remains extremely challenging to determine the precise metabolic status of the bacilli during different stages of the infection cycle in vivo. Moreover, infecting populations of *M.tb* bacteria are located in discrete cellular microenvironments in a single infected host [5, 6, 20, 70].

Rather than investigating metabolic function directly and at the level of complete biological system during TB disease, investigations of mycobacterial metabolism have concentrated primarily on elucidating either the metabolic capacity of the bacillus in vitro in defined growth media and under specific environmental conditions or in experimental models of infection [6]. Metabolic related TB potential drug targets were mostly identified using directed mutagenesis techniques and metabolomics. Metabolomics is promising in elucidating other potential drug targets. Metabolomics analyses based on fate of intermediates of central carbon metabolism [6, 20, 69, 71] has revealed central carbon metabolism anti-TB targets. It has been shown that the main difference in metabolism between *M.tb* and other bacteria is the phenomenon of ‘’compartmentalized co-catabolism of carbon substrates’’ [20].

Many bacteria use catabolite repression as a regulatory mechanism to maximize growth by consuming individual carbon substrates in a preferred sequence. Thus growth follows diauxic kinetics defined by phases of growth determined by available substrates. Surprisingly, untargeted metabolite profiling of *M.tb* growing on ^13^C-labeled carbon substrates revealed that *M.tb* could catabolize multiple carbon sources simultaneously to achieve enhanced monophasic growth. Moreover, when co-catabolizing multiple carbon sources, *M.tb* differentially catabolized each carbon source through the glycolytic, pentose phosphate, and/or tricarboxylic acid (TCA) pathways to distinct metabolic fates [20]. Besides the phenomenon of compartmentalization co-catabolism of carbon substrates, *M.tb* was found to reprogram some metabolic pathways to adapt to granulomas. To establish infection, inside the granuloma, *M.tb* reprograms its metabolism to support both growth and survival, keeping a balance between catabolism, anabolism and energy supply [72]. In a study to explore the metabolic reprogramming of two *M.tb* knockout mutants (*pfkA*- and *icl*-mutants), lacking key enzymes (Phosphofructokinase, PFKA and Isocitrate lyase, ICL) of central carbon metabolism, hypoxia increases glucose consumption. Interestingly, *M.tb pfkA*-mutant showed a detrimental growth effect derived from accumulation of toxic sugar phosphate intermediates (glucose-6-phosphate and fructose-6-phosphate) and tricarboxylic acid cycle also decreased. Metabolic reprogramming of the *icl*-mutant (*icl1* & *icl2*) is a probable indicator of the importance of methylmalonyl pathway for detoxification of propionyl-CoA at high fatty acid consumption rates. Elevated levels of fatty acid uptake and hypoxia leads to a drop in TCA cycle intermediate accumulation thus possibly limiting redox imbalance [72].

**Fig. 2:**
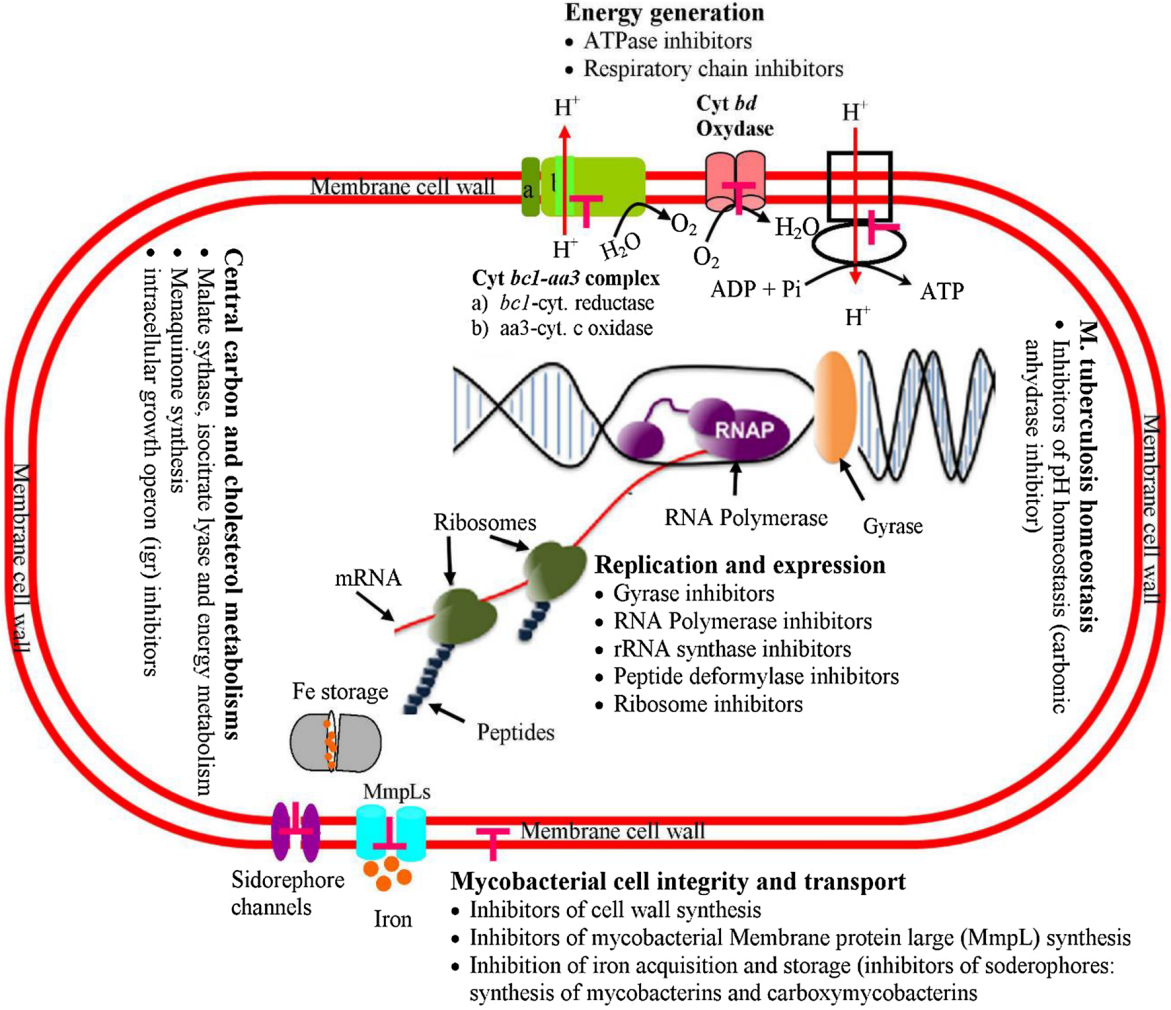
Potential drug targets in *M.tb* metabolism. Various TB drugs exhibit different action mechanisms and target distinct metabolic levels of the *M.tb* cell that are indicated in Figure. Targets of inhibitor drugs are shown below each level

There are many potential drugs targets in *M.tb* metabolism (Fig. 2).

These include iron acquisition and storage, Mycobacterial membrane protein large (MmpL), Cholesterol metabolism, *M.tb* cutinase-like protein Proteases (ClpP) (cutinase-like proteins, Chaperon-linked protease, and caseinolytic proteases), CCM, energy generation, i.e. inhibitors of the respiratory chain and ATP synthesis and reactive oxygen species (ROS) and nitrogen oxygen species (NOS) generation [18].

## Current approaches in TB drug discovery

A global threat posed by TB clearly demonstrates the need to search for new therapeutic regimens with improved outcomes including shorter duration of treatment and sustained efficacy. The following sections discuss the main approaches to achieve the TB drug discovery, production and development.

## Target structure-based high-throughput screening

The first step in drug discovery is the identification of initial chemical leads with reasonable affinity (K_D_ of 10 μM or less) towards the target and novel structures (patentable) that have drug-like characteristics (Lipinski’s Rule of Five) [73], synthetically achievable and malleable [24]. This is primarily accomplished by using a high-throughput screen of a chemical library composed of hundreds of thousands to millions of compounds [24, 74]. The chemical leads are then evolved to high-affinity ligands (K_D_ ≤ nM) through an iterative process involving structure-based drug design, traditional medicinal chemistry techniques, and multiple activity assays. This process may include whole cell-based assays to measure cell viability when the desired activity of a chemical lead is cell death (i.e., infectious disease and cancer) [18, 24, 37]. While this process is generally very efficient and leads to the emergence of high affinity ligands, there is a fundamental difference between a true drug lead and a tight-binding ligand [18, 37]. It is well-known that an increase in affinity can be achieved by increasing the size and hydrophobicity of a compound, which are generally detrimental to drug-like characteristics [24]. Despite significant effort is being expended on *M.tb* target based biochemical screens [18, 75], no TB drug or drug candidate has emerged yet. Problems associated with target-based screens include their propensity to identify hits with potency against the target but without inhibition of bacterial growth. The target inhibition may not translate into bacterial killing for a variety of reasons including poor penetration and efflux [18]. In addition, the lack of efficacy and toxicity issues, which include off-target activity, are the primary reasons for failure of potential drugs in the clinic [24]. Metabolomics is thought to offer solutions that may provide new directions to overturn this limitation.

## Metabolomics approaches and its application to medicinal plants with anti-tuberculosis activity

**Fig. 3:**
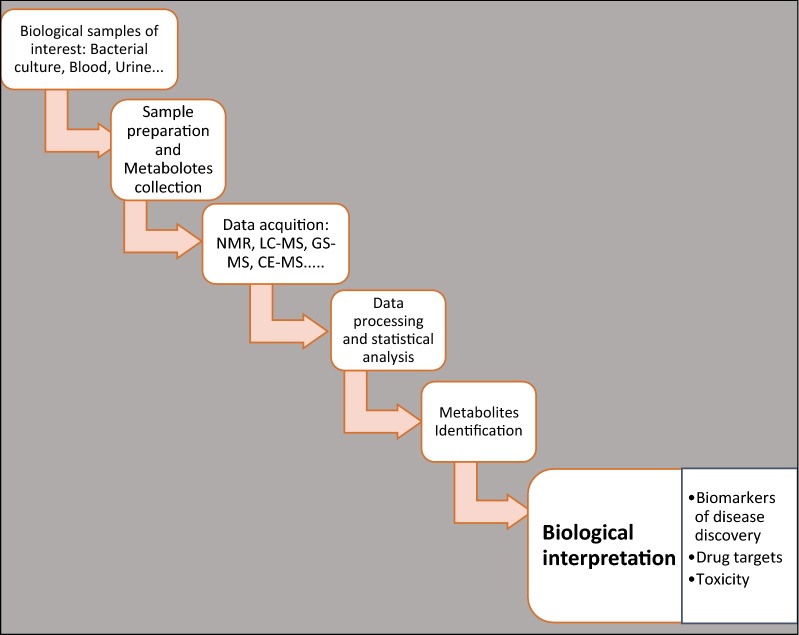
Principles of metabolomics: the biological samples of interest are prepared to stop the metabolism (quenching) and the metabolites are collected. The data can be acquired by different analytical methods. The most common are NMR and Chromatography–MS. The data are processed and statistically analyzed. The metabolites resulting from any fluctuation are identified and biological interpretation gives the meaning of the resulting change

Metabolomics, a high-throughput analysis of metabolites is a recent and emerging type of omics biotechnology. It can be defined as the study of the complete set of small molecules of cells, tissues, organs and biological fluids [29, 76, 77]. Metabolomics is thus considered to be a systematic identification and quantitation of all metabolites in a given organism or biological sample using a range of analytical tools to scale the enormously large data generated. Further, metabolomics depends on molecular detection systems, statistics and bioinformatics (Fig. 3). It represents a major and rapidly evolving component of a systems biology [29, 76, 77], which is an integrative computational analysis and modeling of an organism and its well-being [28, 29, 78, 79]. With metabolomics, (i) metabolic profile, (ii) metabolic fingerprint and (iii) metabolic footprinting of an organism can be determined [31, 80]. As stated above, metabolomics can overcome the challenges related to drug discovery and development. Because the metabolome captures the state of the cell and is a direct measure of protein activity, any observed changes in the metabolome as a result of a drug treatment would provide information on the drug’s activity and selectivity [5, 30, 69, 70].

**Fig. 4:**
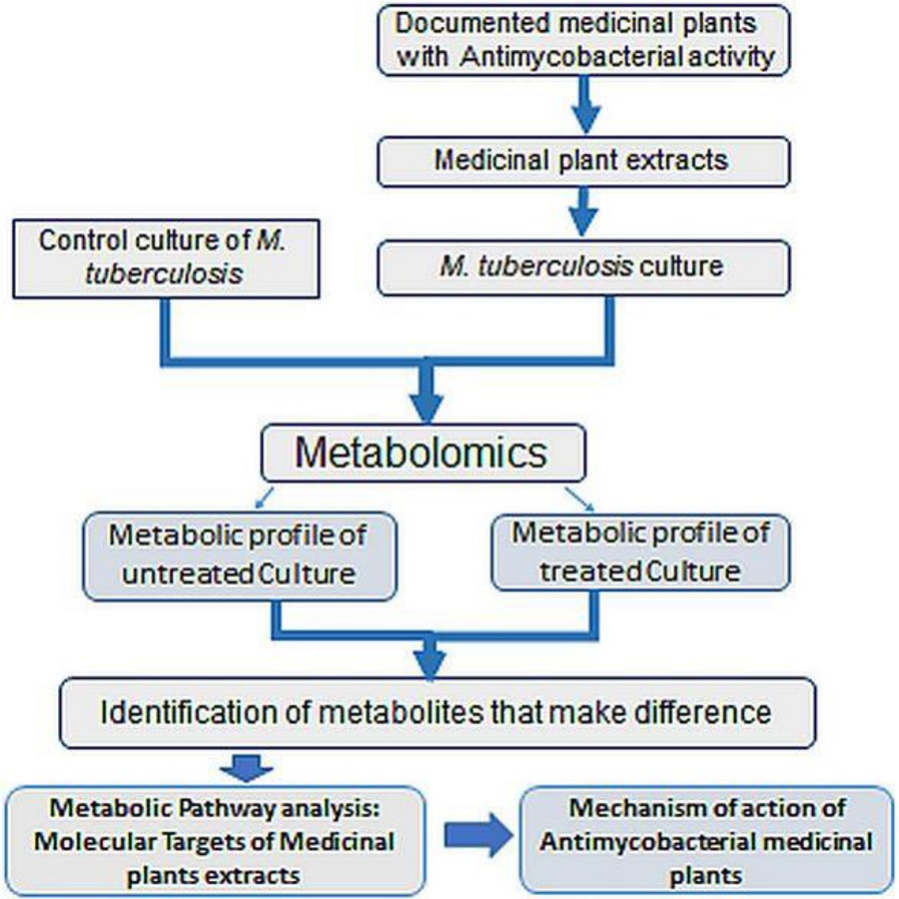
Application of metabolomics to understanding the mechanisms of action of medicinal plants with anti-tuberculosis activity: The metabolic profile of the *M.tb* culture with or without the treatment with antimycobacterial medicinal plant extracts is determined by applying Metabolomics. The change in metabolome of the culture with the treatment is caused to the activity of plants extracts. The metabolites that make difference between the treated and untreated cultures will allow to elucidate the metabolic targets of medicinal plants

The in vivo activity of a chemical lead can then be ascertained by metabolomics. Three samples are needed: (i) untreated wild-type cells representing a negative control, (ii) a knockout mutant cell line, where a gene encoding a target protein has been genetically inactivated, serving as a positive control and (iii) treated wild-type cells as test sample. The metabolome of the mutant and treated wild-type cells would show similar profile [24, 81]. Metabolomics may hence be exploited to identify potential novel anti-*M.tb* candidates and their targets from medicinal plants and elucidate the mechanisms thereof as well (Fig. 4). The complete exercise of drug discovery, development and production is tedious and involves multiple steps. The whole process from the drug screening, through candidate validation, to clinical testing and eventual clinical adoption would lucidly require understanding the biochemical system of the disease itself a systems, pharmacological properties (i.e. absorption, distribution, metabolism, excretion, and toxicity—ADMET) of the therapeutic agents and their functional effects on the human body, both on-target and off-target actions [31, 82]. Despite the fact that metabolomics is increasingly being adopted in drug discovery programs, there are currently no report on the use of the approach to unravel the action mechanisms of medicinal plants with anti-mycobacterial activities; though these have been extensively documented [17, 18, 37, 83–88]. There is a need to establish a suitable metabolomics protocol to reveal the mechanisms of action of plant-derived antimycobacterial agents. The protocol would presumably offer an advantage to identify both TB targets and the chemical lead simultaneously. Protocols for NMR analysis of bacterial metabolome were thoroughly reviewed in the past [89]. However, there are no reports on extension of the approach in the attempt to understand the action mechanisms anti-mycobacterial activity from medicinal plants. On the other hand, other analytical techniques such as chromatographic mass spectrometry (MS) may be used to generate metabolomics data as an alternative to NMR spectroscopy.

In context the of metabolomics application in discovery of new TB drugs, existing protocols can be modified and a variety of procedures might be adopted. For instance (Fig. 4), extracts from the medicinal plants can be screened for activity by adding them into the *M.tb* culture. The level of microorganism growth inhibition would be attributed to the metabolites present in the extracts. To achieve this, there is a need to empirically determine a sub lethal dose from the minimum inhibitory concentration (MIC) of the plants extracts. The dose required here is that translating into a significant change in the metabolome of bacterial cells, but does not reach the lethal concentration. In addition, the metabolic fingerprints of the bacterial culture would reveal the metabolic pathways affected by treatment with the plant extracts. As metabolic pathways are performed by genes products (enzymes), the macromolecules that are compromised by the plant extracts would be identified as well. These macromolecules would be considered as potential target of active compounds present in the extracts that would eventually be developed into new drugs. On the other hand, the metabolomics techniques can be used to identify the active molecules from the plants extracts. Those active molecules are the leads compounds for the drug development. With bioinformatics and other omics technologies such as genomics and transcriptomics, the relationship between identified metabolite or its intermediate products and genes responsible for its production may be elucidated [31, 37, 90, 91]. Thereafter, a large-scale production of the metabolite drug candidate would be envisaged using biotechnology possible applications. Although dramatic improvements in chemotherapy for TB have been achieved through careful studies of drug regimens, there is still a need for new agents exhibiting higher activity. Anti-mycobacterial drugs used at present in therapy for TB were obtained by either blind screening or chemical modification of active compounds [37]. Other approaches based on the knowledge of the biochemistry of the mycobacterial cell should be tried. Certain constituents of the cell, such as mycolic acids, arabinogalactan, peptidoglycan and mycobactin, may represent specific targets for new anti-TB drugs [37, 92]. Many compounds that inhibit specific steps in either arabinogalactan or mycolic acid biosynthesis have been discovered [93]. Novel efficacious and safe anti-TB drugs are currently needed so as: to shorten the duration of TB therapy; treat MDR, XDR and totally drug resistant (TDR) TB strains; and latent TB; act synergistically with other co-administered anti-TB drugs; and, finally, to be safely co-administered with anti-HIV agents [37].

Collectively, there is the possibility of applying metabolomics to identify the macromolecules associated with microbe virulence and their inhibitors. This would likely contribute in the development of new pharmaceuticals with a high selectivity towards their specific targets. In this regard, a well-established protocol would apply and adapt available metabolomics analytical and data analysis techniques and tools to TB drug discovery, development and production. Similarly, this approach could be further extended to other burden human diseases including other infectious diseases and various cancers.

## Conclusion and future perspectives

Human tuberculosis (TB) is one of the oldest and deadliest human bacterial diseases. It is still affecting and posing major health, social and economic burden at the global level. However, most affected people are mainly in low and middle income countries. TB exists in both active and latent, with one-third of the world population being latently infected. Anti-TB drugs with specific mechanisms of action were discovered. Despite this, the length, the cost, the emergence of *Mycobacterium tuberculosis* resistant strains and post-treatment relapse pose serious threats the disease elimination and there is an urgent need of approaches to develop new and more effective news drugs. Target structure-based high-throughput screening for anti-TB drugs leads has been used before. The lack of complete knowledge of *M.tb* biology and the complex *M.tb* pathogenesis have led to the failure. The metabolomics approach is likely to provide new insights into the discovery through understanding their mechanism of action and eventual development of anti TB drugs especially from medicinal plants. To render this feasible, the mechanisms action of the plant-derived antimycobacterial agents should be clearly understood. Metabolomics protocol has to be established in such way as to identify the metabolites affected by antimycobacterial drug leads from the plant extract screened that will guide elucidation of their targets and the mechanisms of their actions.

## Abbreviations

ADMET: absorption, distribution, metabolism, excretion, and toxicity
ATP: adenosine triphosphate
CCM: central carbon metabolism
CE–MS: capillary electrophoresis mass spectrometry
ClpP: cutinase-like protein proteases
CS: cycloserine
DNA: deoxyribonucleic acid
EMA: European Medicines Agency (EMA)
EMB: ethambutol
ETH: 2-ethylisonicotinamide
FAD: Food and Drug Administration
FQ: fluoroquinolone
GEM: genome-scale model
GS–MS: gas chromatography mass spectrometry
HIV: human immunodeficiency virus
ICL: isocitrate lyase
INH: ionicotinylhydrazide
LC–MS: liquid chromatography mass spectrometry
*M.tb*: *Mycobacterium tuberculosis*
MDR: multidrug resistant
MIC: minimum inhibitory concentration
MmpL: Mycobacterial membrane protein large
NAD: nicotinamide adenine dinucleotide
NMR: nuclear magnetic resonance
NOS: nitrogen oxygen species
NRP: non-replicating persistent
PAS: paraaminosalicylic acid
PFKA: phosphofructokinase
PZA: pyrazinamide
RIF: rifampicin
RNA: ribonucleic acid
ROS: reactive oxygen species
RpoB: beta RNA polymerase
SM: streptomycin
TB: tuberculosis
TCA: tricarboxylic acid
TDR: total drug resistant
TMC-207: bedaquiline
WHO: World Health Organization
XDR: extended drug resistant
